# Moral or Legal Responsibility?

**Published:** 1919-03-15

**Authors:** 


*To the Editor of* The Hospital.

### MORAL OR LEGAL RESPONSIBILITY?

*To the Editor of* The Hospital.

Sir,—Which is higher—moral responsibility or legal? Of course we all know that legal responsibility holds the field, but that does not make it moral. Let me come to the point. In order to release "eligibles" for the Army during the past two or three years there were many who gave up permanent positions to take temporary ones—*e.g.*, acting secretaryships of hospitals—and we would gladly do so again for the cause of King and country. But what do we find? Many of those "eligibles" have never been "across," but have spent their time in clerical work "at home." They are now demobilised and return to their safe positions, receiving, as I am informed, a pension in addition to their salary. Let me say that I do not complain about this in the least. But I do ask, what of those who have "carried on" for them during their absence? Are we not entitled to some consideration at the hands of the Government, or at the hands of hospital authorities? Yet we find we are shut out, as witness the many advertisements by the Ministry of Pensions for local secretaries and assistants. Is this fair? Is it British justice? Many will say, "Oh! you knew it was only temporary when you took it on; then why complain?" My reply to that would be, "Every man who enlisted and went to fight knew that he was likely to be killed"; and then I ask if these same people would argue that those Tommies and their dependants were not entitled to consideration? It would be just as reasonable to do so. Fancy men having nothing to complain about when they are shut out from employment! If this kind of talk goes on much longer, the Government had better make up its mind to increase our lunatic asylums for those "beautifully logical" individuals!

I enclose my name and address in "strict confidence," together with recent testimonials. Can anything be done?

"Justice."

[The answer to "Justice" is: Refer to the Parable of the Vineyard and master it speedily.—Ed. *T.H.*]

